# Supplementary material for: Early warning signals have limited applicability to empirical lake data
Source: Nat Commun. 2023 Dec 1;14:7942. doi: 10.1038/s41467-023-43744-8 (PMC10692136; doi:10.1038/s41467-023-43744-8)
Supplement: Supplementary file 1 — Supplementary information [file 41467_2023_43744_MOESM1_ESM.pdf]

Supplementary Information: Early warning signals have limited applicability to empirical lake data.

Duncan A. O'Brien [a*], Smita Deb [b], Gideon Gal [c], Stephen J. Thackeray [d], Partha S. Dutta [b], Shin-ichiro S. Matsuzaki [e], Linda May [f], Christopher F. Clements [a]

[a] School of Biological Sciences, University of Bristol, Bristol, BS8 1TQ, UK
[b] Department of Mathematics, Indian Institute of Technology Ropar, Rupnagar, Punjab 140001, India
[c] Kinneret Limnological Laboratory, Israel Oceanographic & Limnological Research, PO Box 447, Migdal, Israel
[d] Lake Ecosystems Group, UK Centre for Ecology & Hydrology, Bailrigg, Lancaster, UK
[e] Biodiversity Division, National Institute for Environmental Studies, 16-2 Onogawa, Tsukuba, Ibaraki, 305-8506, Japan
[f] UK Centre for Ecology & Hydrology, Bush Estate, Penicuik, Midlothian EH26 OQB, UK

* Duncan A. O'Brien
Email: duncan.a.obrien@gmail.com

Contents

Figure S1: Lake regime shift classification using threshold generalised additive models and kernel densities.
Figure S2-S7: Posterior parameter estimates and diagnostic chain trace plots for Bayesian models.
Figure S8-S10: Diagnostic posterior predictive checks for Bayesian models.
Table S1: Lake breakpoints and bimodality identified by Figure S1.
Table S2: Description of early warning signal indicators used.
Table S3-S7: Model summary tables for the estimated improvement of data pre-processing on early warning signal ability in critically transitioning time series.
Table S8-S9: Model summary tables for early warning signal computation technique ability (i.e. univariate vs multivariate, rolling vs expanding windows) in yearly and monthly data.
Table S10-S11: Model summary tables for individual early warning signal indicator ability (in yearly and monthly data) in predicting critical transitions.
Table S12-S13: Model summary tables for individual early warning signal indicator ability (in yearly and monthly data) in predicting non-critically transitioning time series.

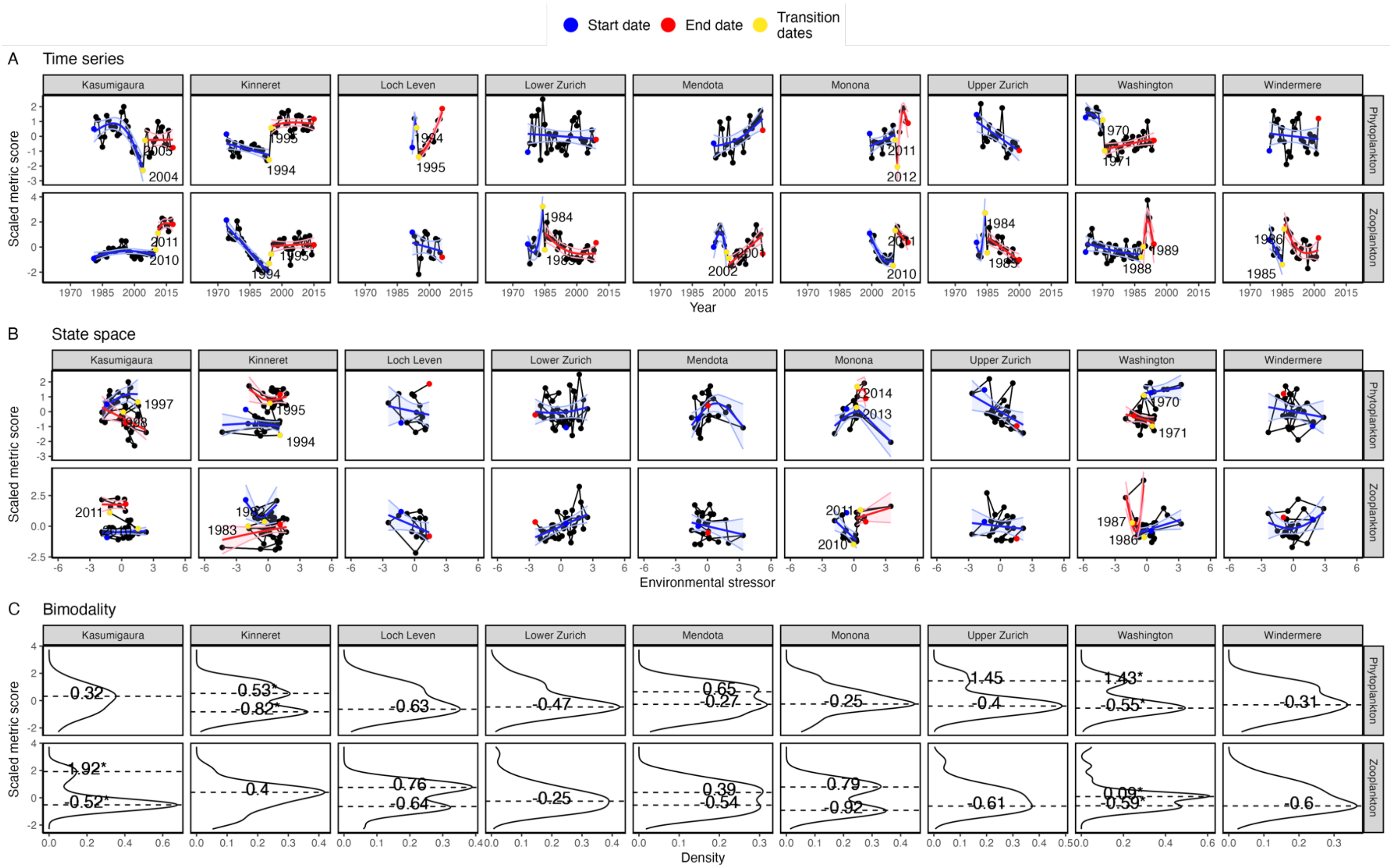

Figure. S1. All threshold generalized additive model fits across lakes and trophic levels. Black points and lines represent the raw time series of plankton density in both the temporal and environmental state spaces. Start, end, and transition points are indicated by coloured points, with the dates of breakpoints also reported. Points represent the observed data, with curved lines and shaded regions the GAM fits and 95% confidence intervals respectively. Asterisks in the kernel density plot indicate significant bimodality coefficients, with dashed lines the estimated modalities. Plankton densities have been scaled to mean zero and unit variance to improve plotting clarity.

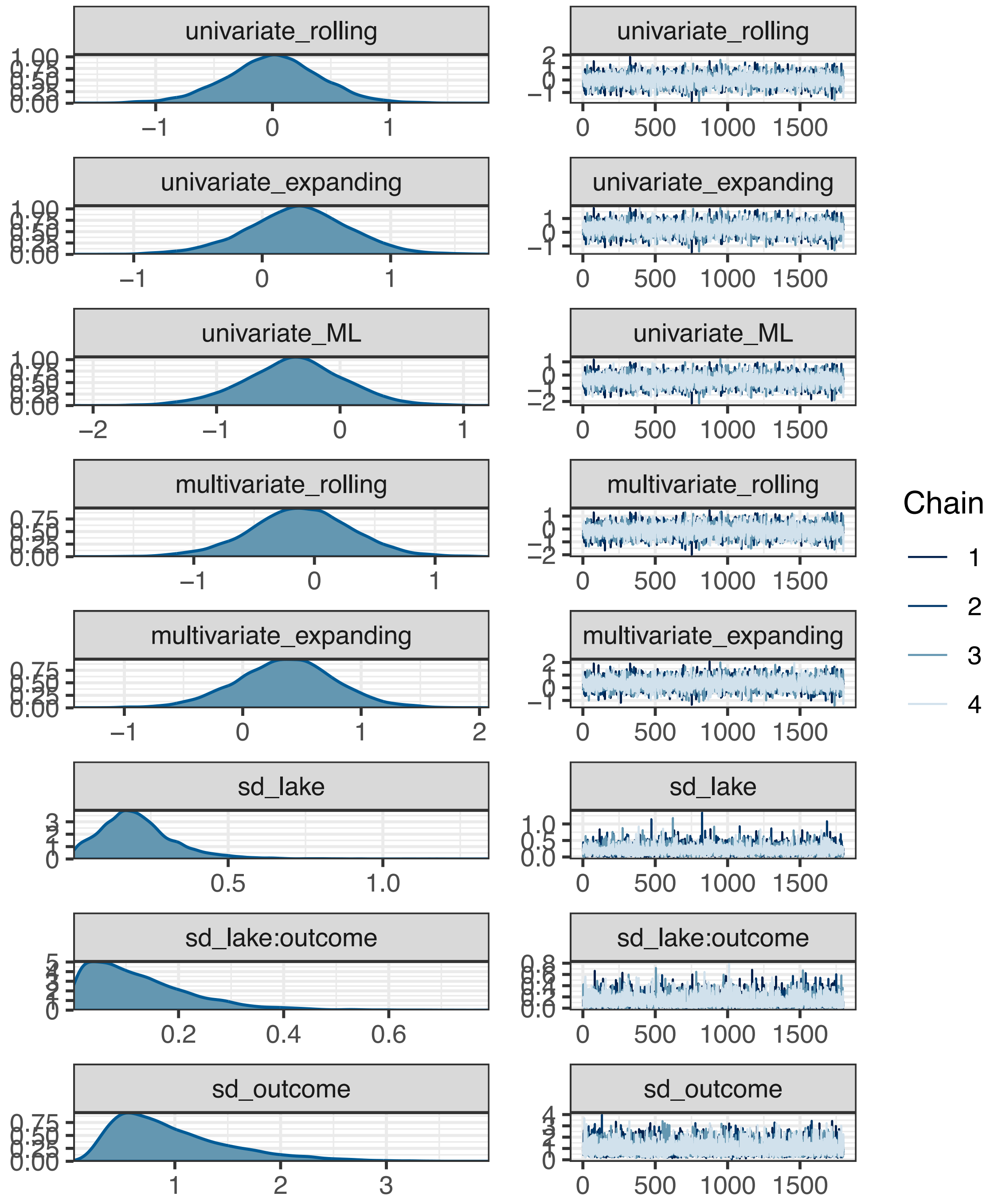

Figure. S2. Trace plots for each parameter of a hierarchical binomial Bayesian model fitted between early warning signal computation method and successful prediction of lake fate in monthly plankton data. Visually, a converged fit is indicated by unimodal density plots (left) and 'well-mixed'/highly overlapping chains (right).

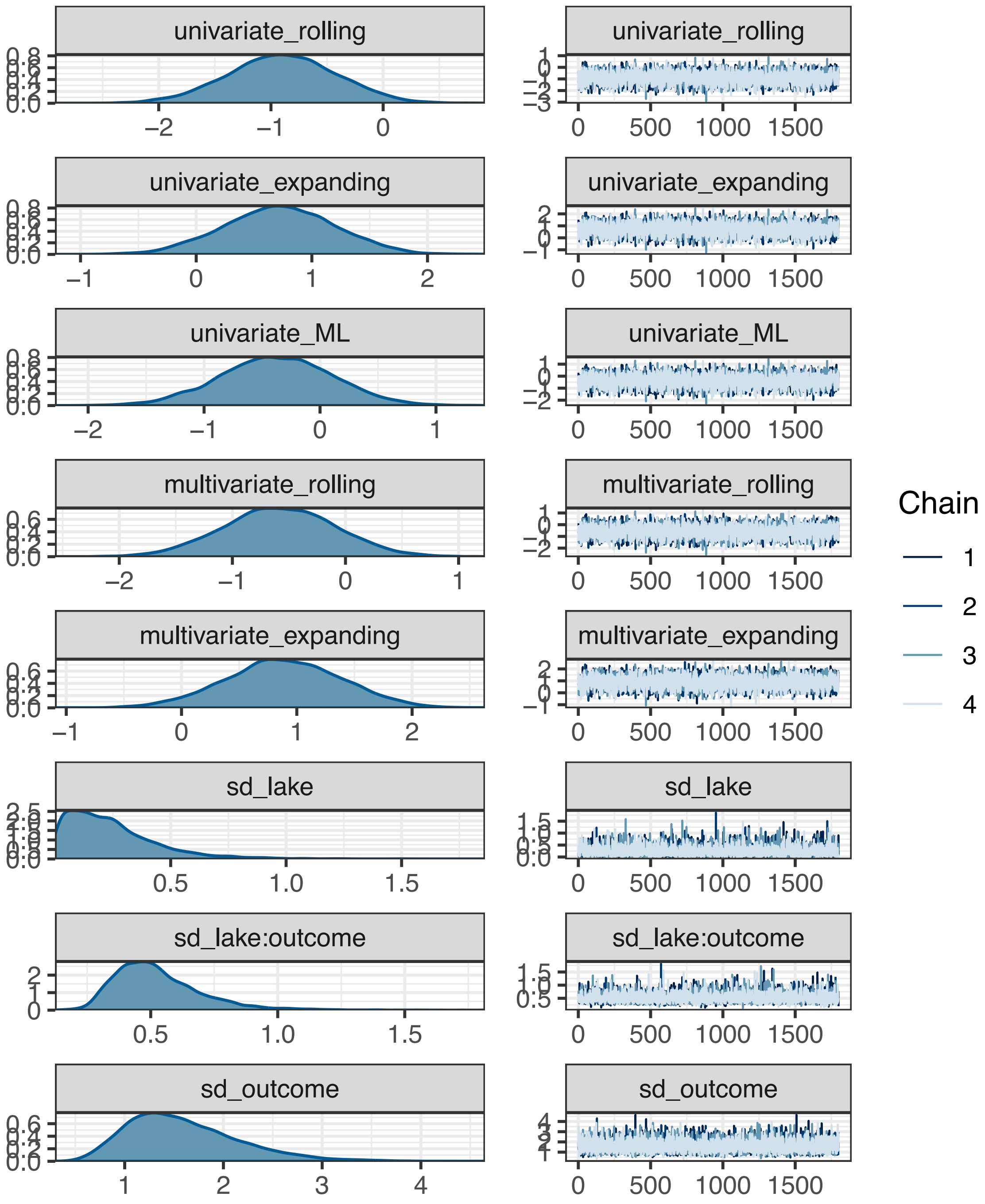

Figure. S3. Trace plots for each parameter of a hierarchical binomial Bayesian model fitted between early warning signal computation method and successful prediction of lake fate in yearly plankton data. Visually, a converged fit is indicated by unimodal density plots (left) and 'well-mixed'/highly overlapping chains (right).

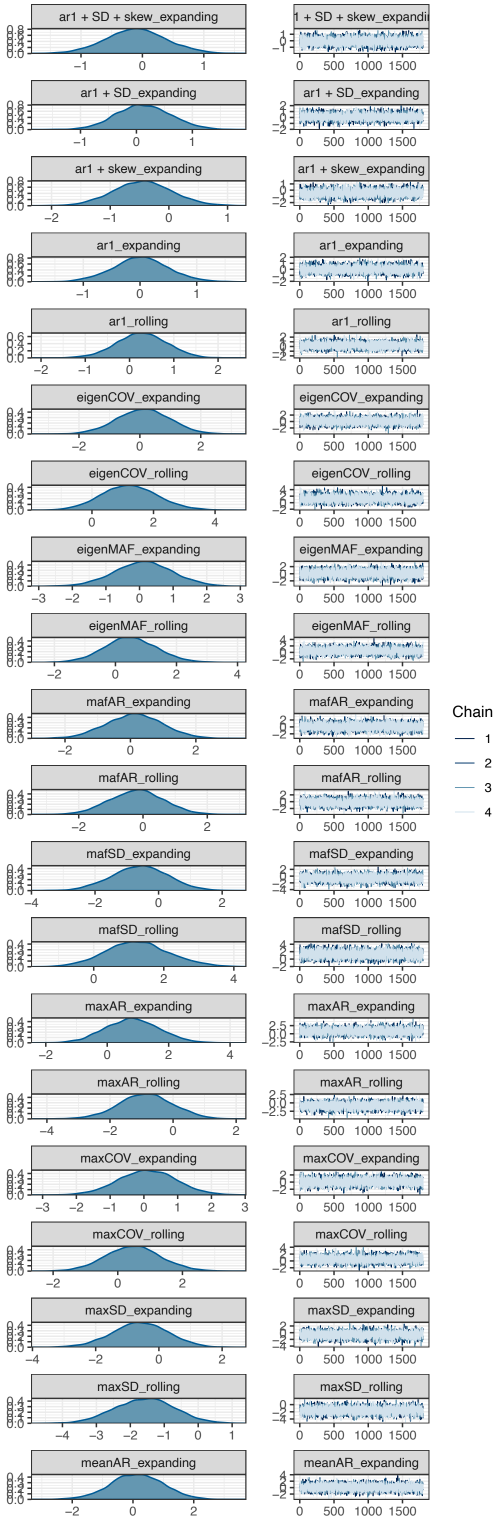

Figure. S4. Trace plots for each parameter of a hierarchical binomial Bayesian model fitted between early warning signal indicator and successful prediction of lake fate in transitioning monthly plankton data.

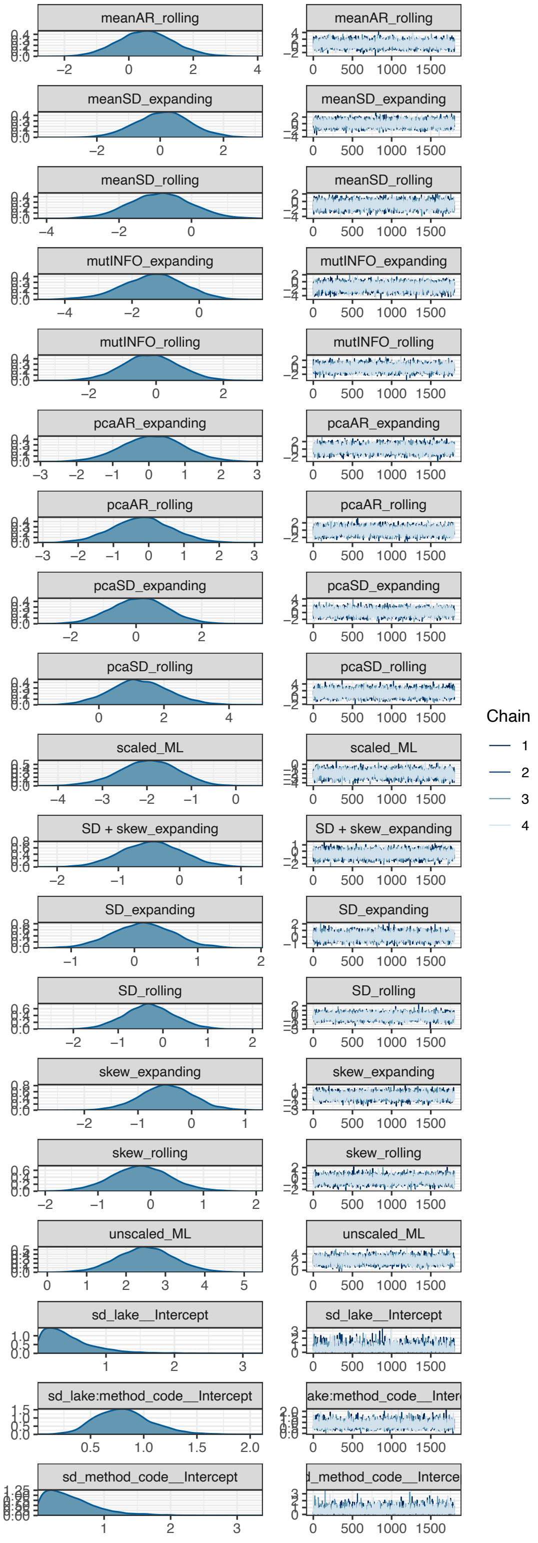

Figure. S4 cont.

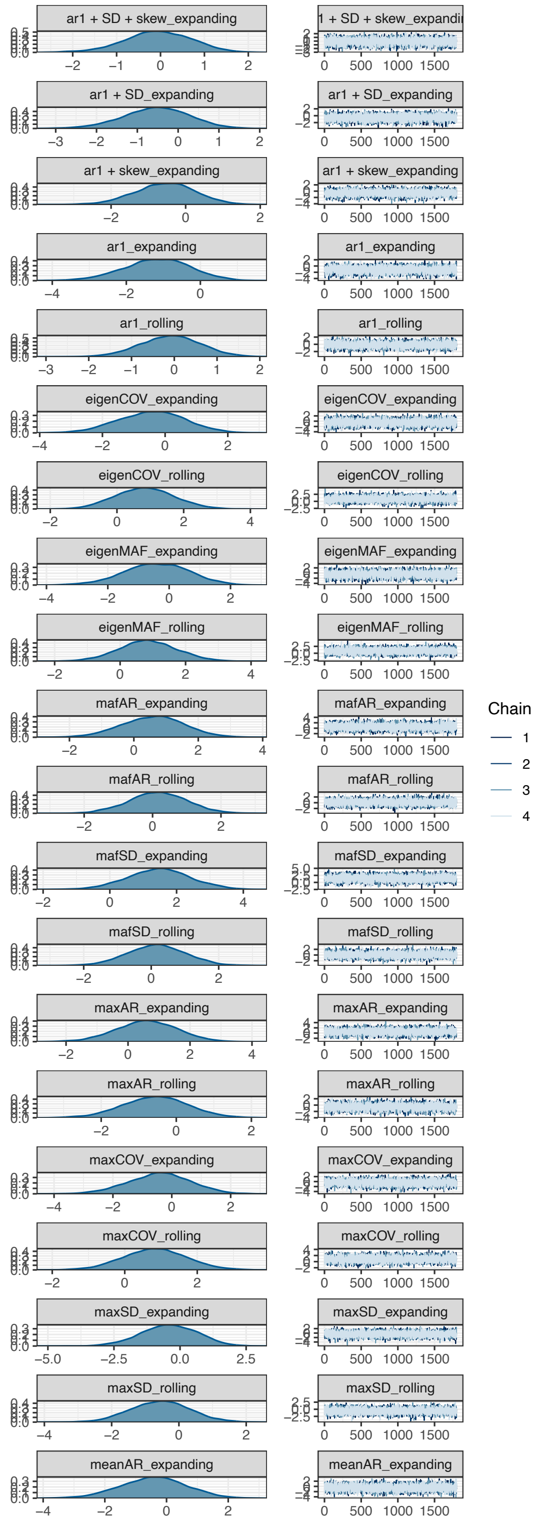

Figure. S5. Trace plots for each parameter of a hierarchical binomial Bayesian model fitted between early warning signal indicator and successful prediction of lake fate in transitioning yearly plankton data.

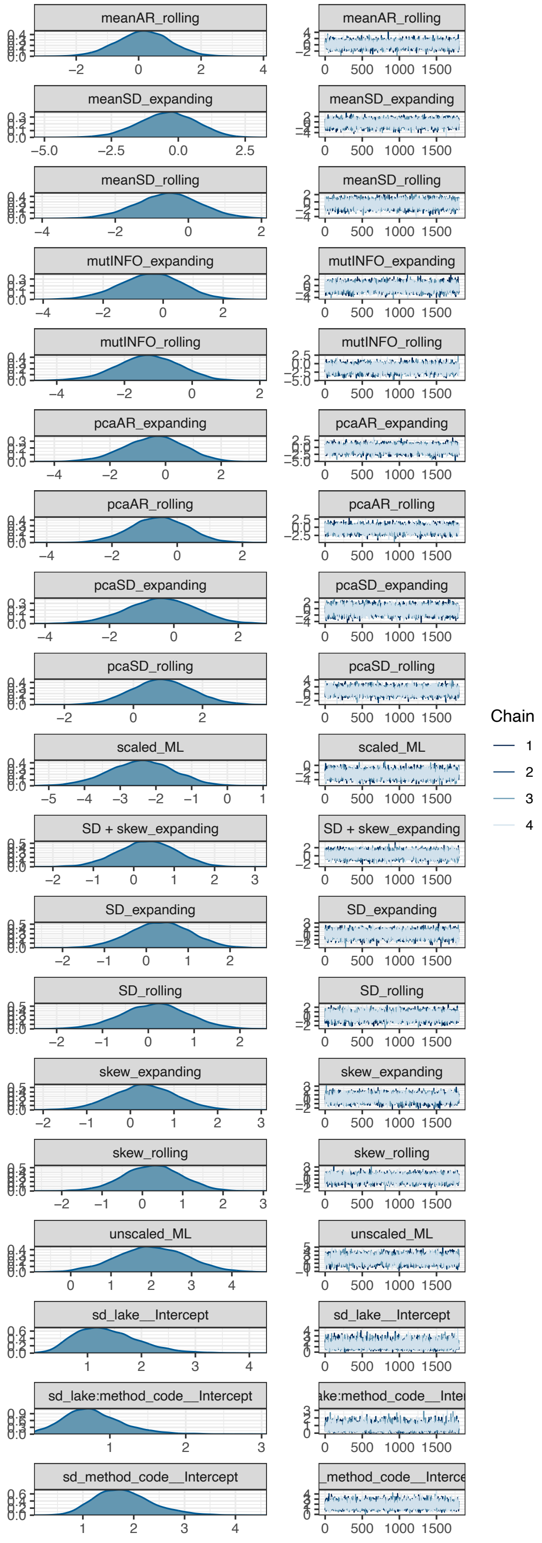

Figure. S5 cont.

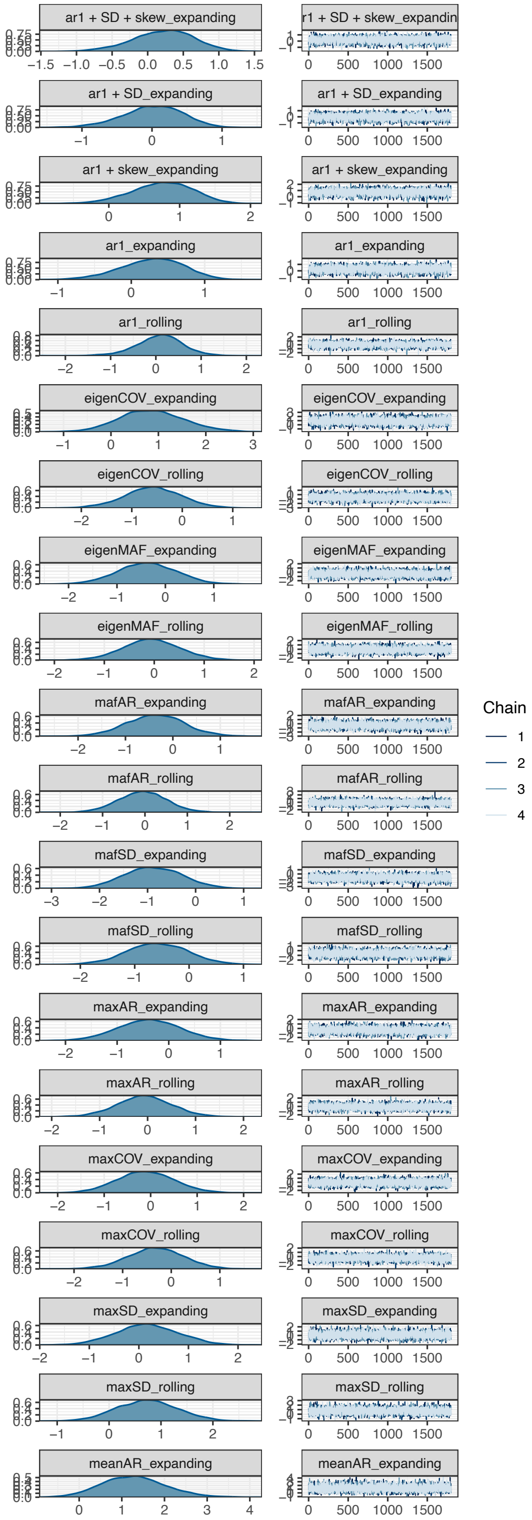

Figure. S6. Trace plots for each parameter of a hierarchical binomial Bayesian model fitted between early warning signal indicator and successful prediction of lake fate in non-transitioning monthly plankton data.

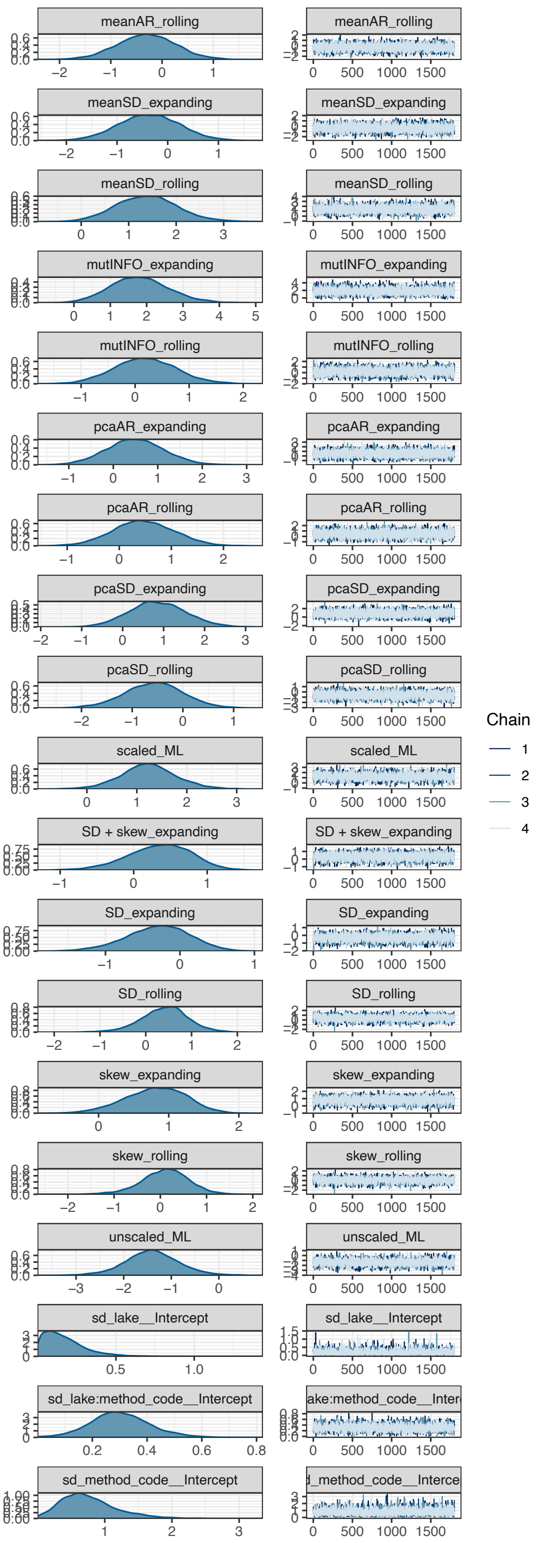

Figure. S6 cont.

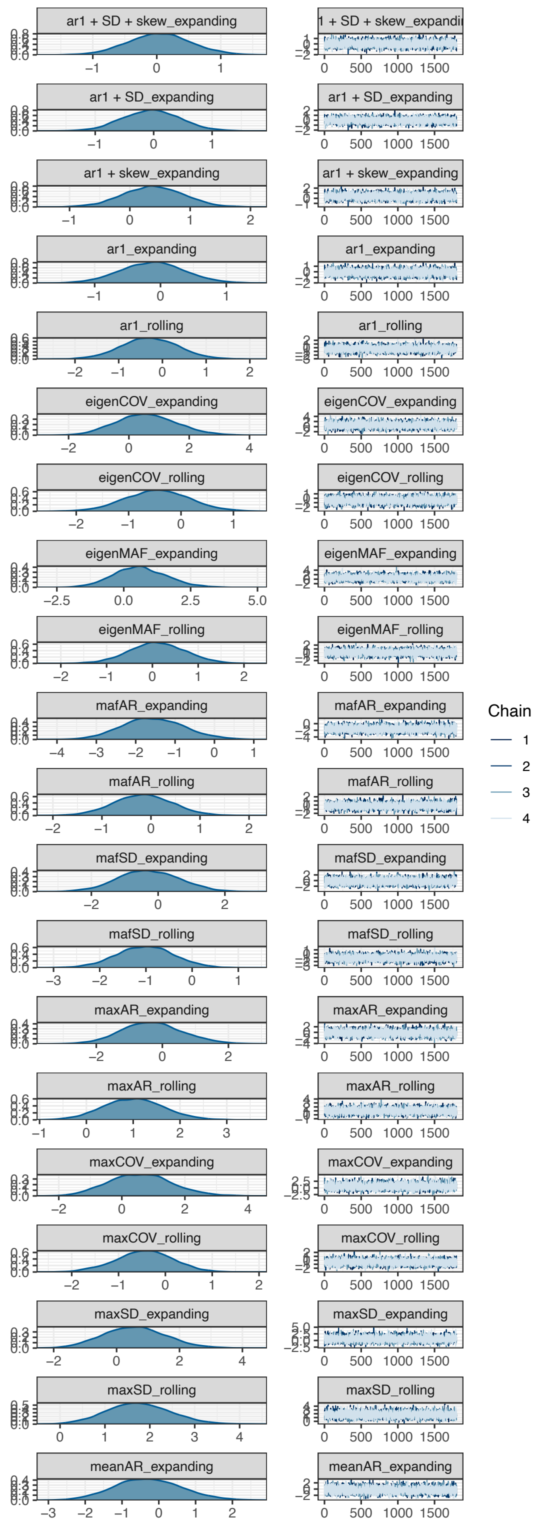

Figure. S7. Trace plots for each parameter of a hierarchical binomial Bayesian model fitted between early warning signal indicator and successful prediction of lake fate in non-transitioning yearly plankton data.

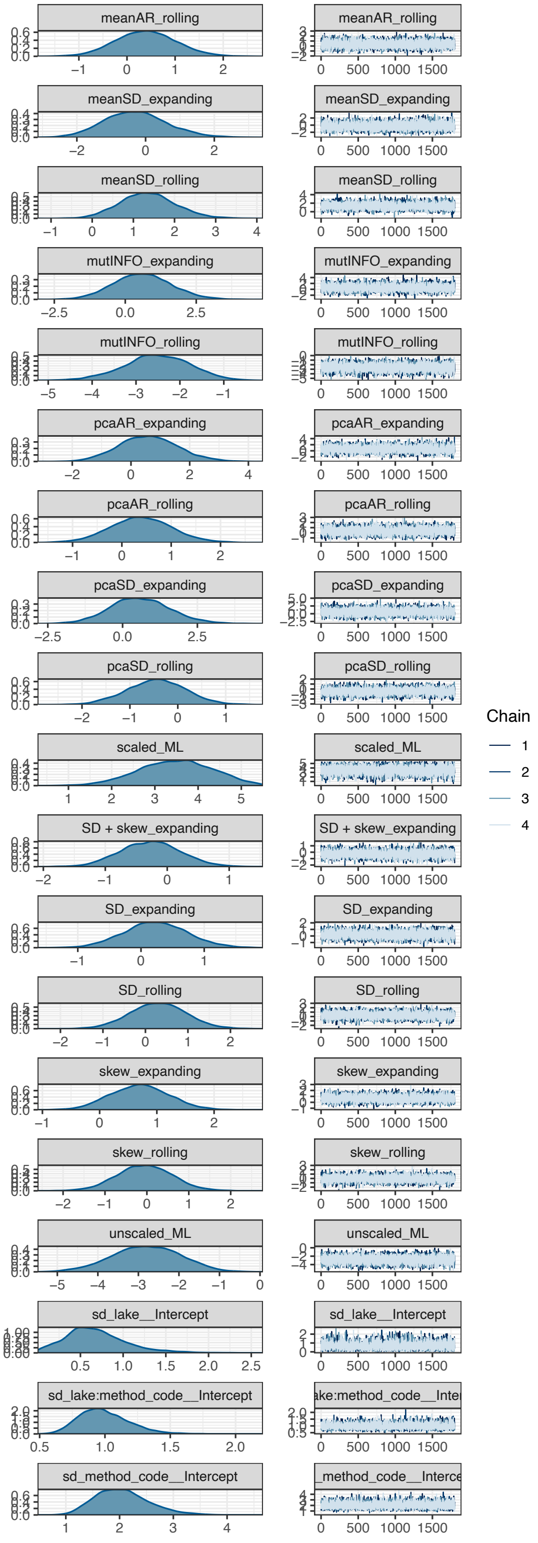

Figure. S7 cont.

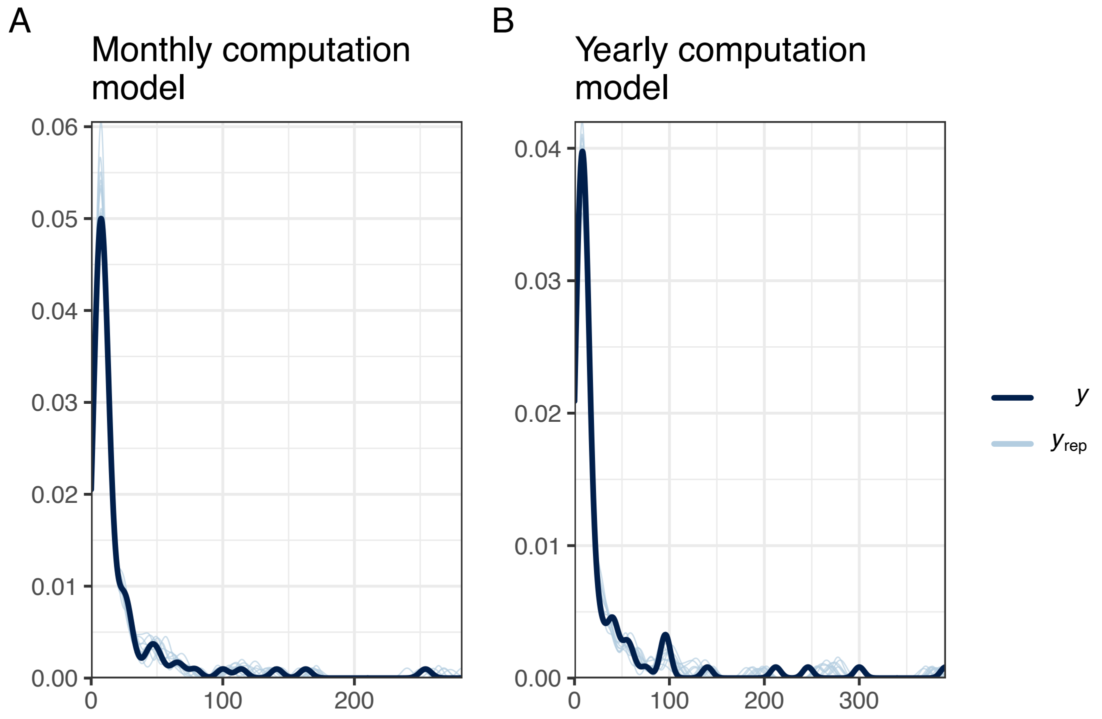

Figure. S8. Posterior predictive checks of hierarchical binomial Bayesian models fitted between early warning signal computation method and successful prediction in A) monthly and B) yearly plankton data. An appropriate fit occurs when *yrep* reasonably reflects *y*.

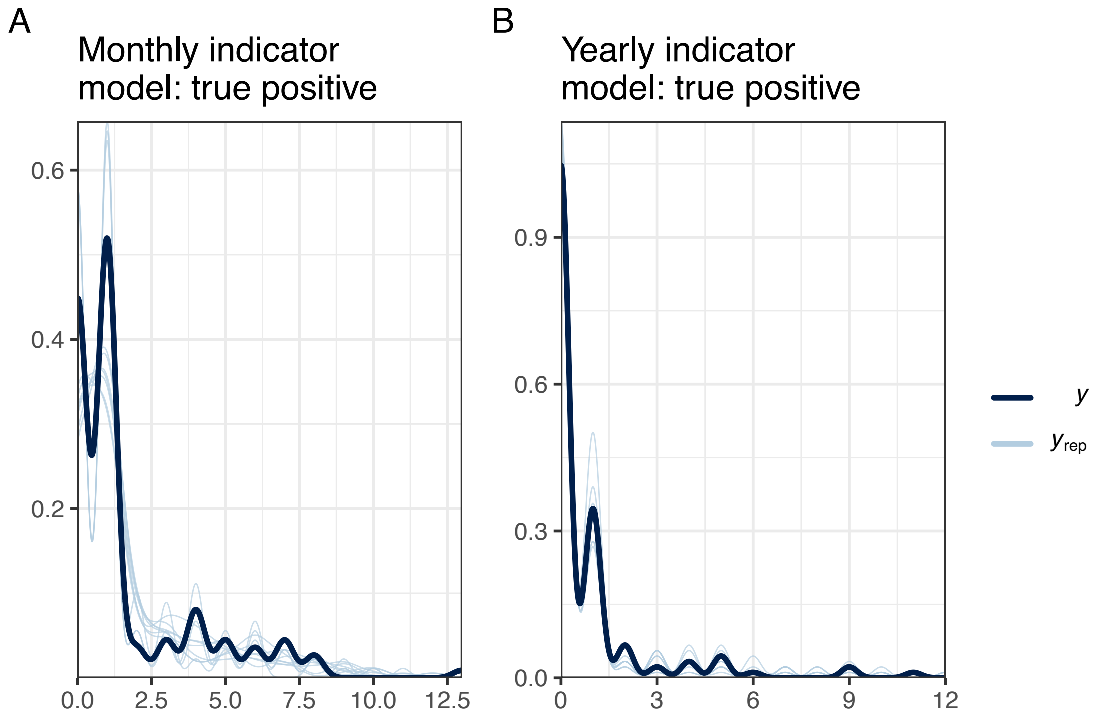

Figure. S9. Posterior predictive checks of hierarchical binomial Bayesian models fitted between early warning signal indicator and successful prediction in A) monthly and B) yearly transitioning plankton data. These models represent the true positive ability of each indicator. An appropriate fit occurs when *yrep* reasonably reflects *y*.

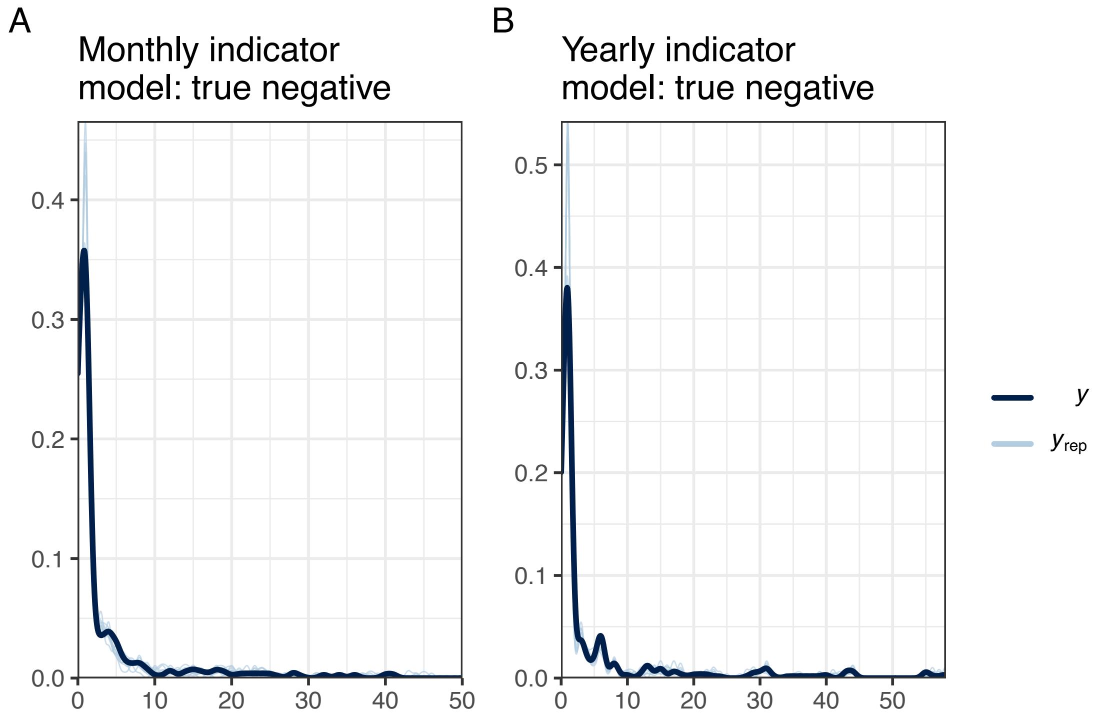

Figure. S10. Posterior predictive checks of hierarchical binomial Bayesian models fitted between early warning signal indicator and successful prediction in A) monthly and B) yearly non-transitioning plankton data. These models represent the true negative ability of each indicator. An appropriate fit occurs when *yrep* reasonably reflects *y*.

Table S1. Breakpoints and critical transitions identified from threshold generalised additive models.

Lake	Trophic level	Explanatory variable	Breakpoint year	Bimodality detected	Critical transition identified
Kasumigaura	Phytoplankton	Time	2004	No	No
		Environment	1997		
	Zooplankton	Time	**2010**	**Yes**	**Yes**
		Environment	**2010**		
Kinneret	Phytoplankton	Time	**1994**	**Yes**	**Yes**
		Environment	**1994**		
	Zooplankton	Time	1982	No	No
		Environment	1994		
Loch Leven	Phytoplankton	Time	1994	No	No
		Environment	NA		
	Zooplankton	Time	NA	No	No
		Environment	NA		
Lower Zurich	Phytoplankton	Time	NA	No	No
		Environment	NA		
	Zooplankton	Time	1984	No	No
		Environment	NA		
Mendota	Phytoplankton	Time	NA	No	No
		Environment	NA		
	Zooplankton	Time	2001	No	No
		Environment	NA		
Monona	Phytoplankton	Time	2011	No	No
		Environment	2013		
	Zooplankton	Time	**2010**	**Yes**	**Yes**
		Environment	**2010**		
Upper Zurich	Phytoplankton	Time	NA	No	No
		Environment	NA		
	Zooplankton	Time	1984	No	No
		Environment	NA		
Washington	Phytoplankton	Time	**1970**	**Yes**	**Yes**
		Environment	**1970**		
	Zooplankton	Time	1988	Yes	No
		Environment	1986		
Windermere	Phytoplankton	Time	NA	No	No
		Environment	NA		
	Zooplankton	Time	1985	No	No
		Environment	NA		

Table S2. Description of each individual early warning signal indicator and which variate category it belongs to.

Early warning signal method	Indicator	Description	Abbreviation
Univariate	autocorrelation at lag-1	The similarity between temporally adjacent data points – i.e. the correlation between the time series and the lagged version of itself.	ar1
	variance	A measure of the degree of dispersion displayed in the time series. Is represented as the standard deviation here.	SD
	skewness	The degree of asymmetry in the distribution of values displayed in the time series.	skew
	composite of each combination of the above	In the expanding window computation, the three above indicators can be standardised and combined to improve their reliability. Two and three indicator combinations were performed in this study.	ar1 + SD, ar1 + skew, SD + skew, ar1 + SD + skew
Multivariate	mean autocorrelation at lag-1	Average autocorrelation across all time series representing the system.	meanAR
	max autocorrelation at lag-1	The strongest autocorrelation of all time series representing the system.	maxAR
	mean variance	Average standard deviation across all time series representing the system.	meanSD
	max variance	The largest standard deviation of all time series representing the system.	maxSD
	min/max autocorrelation factor (MAF) dominant eigenvalue	Following MAF dimension reduction of all representative time series, smallest scalar	eigenMAF

		of the resulting eigenvectors.	
	first MAF (MAF1) autocorrelation at lag-1	The autocorrelation of the MAF axis that yields the strongest autocorrelation.	mafAR
	MAF1 variance	The standard deviation of the MAF axis that yields the strongest autocorrelation.	mafSD
	first principal component (PC1) autocorrelation at lag-1	Following principal component analysis of all representative time series, the autocorrelation of the principal component which explains the greatest variance.	pcaAR
	PC1 variance	The standard deviation of the first principal component.	pcaSD
	dominant eigenvalue of the covariance matrix	From the covariance matrix of all representative time series, the largest eigenvalue is informative.	eigenCOV
	maximum covariance	The strongest covariance between all representative time series.	maxCOV
	mutual information	The degree of information gained from one time series on the state of another. Is averaged across all pairwise time series comparisons.	mutINFO
Machine learning	EWSNet scaled weights	Calls the scaled forms of the EWSNet model weights (scaled between 1-2) and conceptually should be robust regardless of the data’s magnitude.	scaled
	EWSNet unscaled weights	Calls the unscaled forms of the EWSNet model weights.	unscaled

Table S3. Coefficient estimates for influence of each data pre-processing technique on rolling window univariate early warning signal classification ability relative to assessments made on the raw data. Each estimate is therefore the relative improvement of that factor level versus the none-none data pre-processing. Rhat was equal to 1 for all estimates.

**Pre-processing combination (detrending method – deseasoning method)**	**Estimated improvement relative to no pre-processing (median)**	**Lower 95% credible interval**	**Upper 95% credible interval**	**Effective sample size**
linear-none	0.022	-0.281	0.326	4404.74
loess-none	-0.029	-0.336	0.278	4645.2
gaussian-none	-0.04	-0.349	0.271	4485.97
none-average	0.022	-0.287	0.338	4698.69
none-decomposition	0.05	-0.256	0.368	4436.14
none-stl	-0.018	-0.32	0.295	4483.88
linear-average	0.009	-0.299	0.311	4497.63
loess-average	0.035	-0.274	0.341	4496.95
gaussian-average	0.023	-0.282	0.339	4616.81
linear-decomposition	0.037	-0.273	0.345	4429.23
loess-decomposition	0.009	-0.296	0.315	4933.83
gaussian-decomposition	0.011	-0.298	0.324	4567.6
linear-stl	0.079	-0.223	0.39	4381.49
loess-stl	0.025	-0.282	0.336	4682.63
gaussian-stl	0.041	-0.271	0.346	4460.24

Table S4. Coefficient estimates for influence of each data pre-processing technique on expanding window univariate early warning signal classification ability relative to assessments made on the raw data. Each estimate is therefore the relative improvement of that factor level versus the none-none data pre-processing. Rhat was equal to 1 for all estimates.

**Pre-processing combination (detrending method – deseasoning method)**	**Estimated improvement relative to no pre-processing (median)**	**Lower 95% credible interval**	**Upper 95% credible interval**	**Effective sample size**
linear-none	0.022	-0.191	0.225	4862.99
loess-none	-0.011	-0.219	0.188	4243.27
gaussian-none	-0.047	-0.261	0.156	4358.7
none-average	-0.101	-0.309	0.111	4425.74
none-decomposition	0.156	-0.053	0.372	4881.34
none-stl	-0.082	-0.285	0.129	4688.05
linear-average	-0.023	-0.231	0.182	4641.8
loess-average	-0.005	-0.21	0.2	4752.65
gaussian-average	-0.036	-0.245	0.168	4744.24
linear-decomposition	0.212	0.003	0.417	4765.92
loess-decomposition	0.146	-0.065	0.354	4436.74
gaussian-decomposition	0.11	-0.093	0.32	5118.86
linear-stl	-0.009	-0.211	0.207	4635.29
loess-stl	0.005	-0.2	0.211	4744.39
gaussian-stl	0.01	-0.196	0.221	4525.97

Table S5. Coefficient estimates for influence of each data pre-processing technique on rolling window multivariate early warning signal classification ability relative to assessments made on the raw data. Each estimate is therefore the relative improvement of that factor level versus the none-none data pre-processing. Rhat was equal to 1 for all estimates.

**Pre-processing combination (detrending method – deseasoning method)**	**Estimated improvement relative to no pre-processing (median)**	**Lower 95% credible interval**	**Upper 95% credible interval**	**Effective sample size**
linear-none	0.109	-0.42	0.647	5858.11
loess-none	0.155	-0.377	0.692	5716.95
gaussian-none	0.198	-0.344	0.729	5788.42
none-average	-0.375	-0.912	0.164	5913.66
none-decomposition	-0.291	-0.839	0.245	5769.71
none-stl	-0.194	-0.713	0.332	5565.13
linear-average	-0.199	-0.736	0.321	5946.3
loess-average	-0.068	-0.602	0.467	5412.68
gaussian-average	-0.071	-0.6	0.458	5879.94
linear-decomposition	-0.069	-0.592	0.473	5625.25
loess-decomposition	-0.07	-0.599	0.464	5662.73
gaussian-decomposition	0.149	-0.367	0.7	5808.23
linear-stl	-0.021	-0.539	0.495	5721.08
loess-stl	0.063	-0.477	0.6	5753.3
gaussian-stl	-0.021	-0.576	0.504	5879.66

Table S6. Coefficient estimates for influence of each data pre-processing technique on expanding window multivariate early warning signal classification ability relative to assessments made on the raw data. Each estimate is therefore the relative improvement of that factor level versus the none-none data pre-processing. Rhat was equal to 1 for all estimates.

**Pre-processing combination (detrending method – deseasoning method)**	**Estimated improvement relative to no pre-processing (median)**	**Lower 95% credible interval**	**Upper 95% credible interval**	**Effective sample size**
linear-none	-0.056	-0.587	0.449	5655.71
loess-none	-0.014	-0.529	0.504	4873.96
gaussian-none	0.114	-0.412	0.647	4887.66
none-average	-0.101	-0.633	0.422	5474.2
none-decomposition	0.114	-0.4	0.63	5160.85
none-stl	-0.101	-0.62	0.431	4805.72
linear-average	-0.102	-0.627	0.413	4637.09
loess-average	-0.106	-0.632	0.415	5453.8
gaussian-average	0.203	-0.309	0.736	5393.85
linear-decomposition	-0.27	-0.784	0.256	4786.78
loess-decomposition	-0.407	-0.928	0.117	4535.2
gaussian-decomposition	0.026	-0.492	0.544	5009.93
linear-stl	-0.06	-0.592	0.442	5226.99
loess-stl	-0.226	-0.748	0.294	5430.75
gaussian-stl	0.119	-0.402	0.649	4725.75

Table S7. Coefficient estimates for influence of each data pre-processing technique on machine learning univariate early warning signal classification ability relative to assessments made on the raw data. Each estimate is therefore the relative improvement of that factor level versus the none-none data pre-processing. Rhat was equal to 1 for all estimates.

**Pre-processing combination (detrending method – deseasoning method)**	**Estimated improvement relative to no pre-processing (median)**	**Lower 95% credible interval**	**Upper 95% credible interval**	**Effective sample size**
linear-none	0.074	-0.302	0.444	4107.36
loess-none	0.167	-0.198	0.538	4735.59
gaussian-none	0.206	-0.148	0.573	5220.93
none-average	-0.023	-0.388	0.337	4872.58
none-decomposition	-0.126	-0.494	0.246	4679.61
none-stl	-0.089	-0.464	0.288	4635.03
linear-average	0.093	-0.277	0.459	4766.82
loess-average	0.07	-0.3	0.439	4796.65
gaussian-average	0.172	-0.204	0.542	4708.95
linear-decomposition	0.095	-0.286	0.462	5137.04
loess-decomposition	0.011	-0.343	0.383	4863.16
gaussian-decomposition	0.093	-0.282	0.466	4922.87
linear-stl	-0.19	-0.546	0.196	5022.74
loess-stl	-0.005	-0.385	0.366	4858.9
gaussian-stl	-0.006	-0.383	0.363	4974.07

Table S8. Raw coefficient estimates for the influence of each early warning signal computational approach on correct classification of the monthly lake plankton dataset. Rhat was equal to 1 for all estimates.

Computation method	Coefficient estimate (median)	Lower 95% credible interval	Upper 95% credible interval	Effective sample size
univariate_rolling	0.01	-0.828	0.823	5547.13
univariate_expanding	0.287	-0.543	1.077	5586.69
EWSNet	-0.364	-1.205	0.444	5571.98
multivariate_rolling	-0.109	-0.97	0.718	5684.71
multivariate_expanding	0.372	-0.492	1.202	5815.47

Table S9. Raw coefficient estimates for the influence of each early warning signal computational approach on correct classification of the yearly lake plankton dataset. Rhat was equal to 1 for all estimates.

Computation method	Coefficient estimate (median)	Lower 95% credible interval	Upper 95% credible interval	Effective sample size
univariate_rolling	-0.897	-1.855	0.038	6679.26
univariate_expanding	0.722	-0.215	1.665	6733.57
EWSNet	-0.377	-1.332	0.568	6764.95
multivariate_rolling	-0.584	-1.551	0.38	6720.27
multivariate_expanding	0.884	-0.105	1.864	6839.85

Table S10. Raw coefficient estimates for the influence of each early warning signal indicator on the correct classification of transitioning monthly lake plankton data. Rhat was equal to 1 for all estimates.

Early warning signal indicator	Coefficient estimate (median)	Lower 95% credible interval	Upper 95% credible interval	Effective sample size
ar1 + SD + skew_expanding	-0.101	-1.065	0.91	5699.4
ar1 + SD_expanding	0.123	-0.807	1.112	6556.57
ar1 + skew_expanding	-0.443	-1.427	0.56	6614.13
ar1_expanding	0.014	-0.968	1.004	6654.9
ar1_rolling	0.278	-0.862	1.376	6881.43
eigenCOV_expanding	0.125	-1.603	1.811	7284.44
eigenCOV_rolling	1.206	-0.513	3.046	7559.88
eigenMAF_expanding	0.126	-1.573	1.824	7211.93
eigenMAF_rolling	0.487	-1.174	2.217	7187.18
mafAR_expanding	0.129	-1.569	1.803	7419.59
mafAR_rolling	-0.183	-1.849	1.451	7307
mafSD_expanding	-0.573	-2.306	1.136	7378.08
mafSD_rolling	1.23	-0.465	3.12	6619.4
maxAR_expanding	0.812	-0.881	2.555	7038.13
maxAR_rolling	-0.862	-2.57	0.812	7142.18
maxCOV_expanding	0.103	-1.589	1.802	7043.55
maxCOV_rolling	0.475	-1.174	2.174	7474.18
maxSD_expanding	-0.595	-2.36	1.084	6843.76
maxSD_rolling	-1.556	-3.321	0.129	7216.39
meanAR_expanding	0.127	-1.583	1.803	6829.76
meanAR_rolling	0.523	-1.115	2.249	7479.18
meanSD_expanding	0.114	-1.608	1.821	7296.07
meanSD_rolling	-0.855	-2.556	0.836	7170.59
mutINFO_expanding	-1.33	-3.308	0.367	7121.51
mutINFO_rolling	-0.168	-1.802	1.486	7240.02
pcaAR_expanding	0.123	-1.569	1.813	7205.42
pcaAR_rolling	-0.187	-1.882	1.489	6942.3
pcaSD_expanding	0.114	-1.551	1.793	7363.32
pcaSD_rolling	1.225	-0.491	3.103	7330.97
scaled_ML	-1.953	-3.372	-0.718	7250.73
SD + skew_expanding	-0.453	-1.418	0.542	6682.21
SD_expanding	0.138	-0.817	1.131	6549.57
SD_rolling	-0.312	-1.402	0.814	7039.61
skew_expanding	-0.464	-1.441	0.538	6941.92
skew_rolling	-0.188	-1.28	0.94	6865.29
unscaled_ML	2.5	1.109	3.964	7196.14

Table S11. Raw coefficient estimates for the influence of each early warning signal indicator on the correct classification of transitioning yearly lake plankton data. Rhat was equal to 1 for all estimates.

Early warning signal indicator	Coefficient estimate (median)	Lower 95% credible interval	Upper 95% credible interval	Effective sample size
ar1 + SD_expanding	-0.518	-2.221	0.991	6839.06
ar1 + skew_expanding	-0.533	-2.189	0.985	7186.25
ar1_expanding	-1.151	-3.048	0.562	6360.64
ar1_rolling	-0.078	-1.5	1.292	7120.56
eigenCOV_expanding	-0.404	-2.538	1.635	7119.7
eigenCOV_rolling	0.831	-0.841	2.569	7084.94
eigenMAF_expanding	-0.402	-2.593	1.617	7401.32
eigenMAF_rolling	0.838	-0.886	2.597	7083.83
mafAR_expanding	0.626	-1.345	2.469	6715.11
mafAR_rolling	0.165	-1.52	1.837	7417.58
mafSD_expanding	1.484	-0.419	3.313	7250.88
mafSD_rolling	0.166	-1.543	1.82	7525.29
maxAR_expanding	0.609	-1.334	2.47	7176.4
maxAR_rolling	-0.541	-2.274	1.158	7202.81
maxCOV_expanding	-0.4	-2.574	1.577	7187.44
maxCOV_rolling	0.831	-0.888	2.563	7621.39
maxSD_expanding	-0.401	-2.559	1.58	6985.81
maxSD_rolling	-0.54	-2.277	1.189	7306.98
meanAR_expanding	-0.411	-2.567	1.605	7144.62
meanAR_rolling	0.167	-1.556	1.812	7007.18
meanSD_expanding	-0.384	-2.594	1.6	7208.06
meanSD_rolling	-0.539	-2.309	1.178	7219
mutINFO_expanding	-0.389	-2.52	1.573	7087.32
mutINFO_rolling	-1.293	-3.194	0.41	7394.37
pcaAR_expanding	-0.375	-2.58	1.601	6952.42
pcaAR_rolling	-0.555	-2.297	1.106	7038.82
pcaSD_expanding	-0.389	-2.544	1.623	7548.03
pcaSD_rolling	0.82	-0.874	2.6	7083.83
scaled_ML	-2.398	-4.212	-0.67	6758.17
SD + skew_expanding	0.341	-1.098	1.69	7227.53
SD_expanding	0.343	-1.101	1.701	6977.24
SD_rolling	0.155	-1.309	1.542	7236.72
skew_expanding	0.321	-1.108	1.715	7500.49
skew_rolling	0.26	-1.172	1.622	6755.08
unscaled_ML	2.011	0.307	3.697	6940.17

Table S12. Raw coefficient estimates for the influence of each early warning signal indicator on the correct classification of non-transitioning monthly lake plankton data. Rhat was equal to 1 for all estimates.

**Early warning signal indicator**	**Coefficient estimate (median)**	**Lower 95% credible interval**	**Upper 95% credible interval**	**Effective sample size**
ar1 + SD_expanding	0.024	-0.903	0.781	4124.16
ar1 + skew_expanding	0.767	-0.144	1.538	3783.99
ar1_expanding	0.307	-0.614	1.063	4152.73
ar1_rolling	0.108	-1.024	1.083	6285.93
eigenCOV_expanding	0.845	-0.434	2.285	6389.6
eigenCOV_rolling	-0.581	-1.728	0.518	7082.21
eigenMAF_expanding	-0.378	-1.544	0.784	6108.65
eigenMAF_rolling	-0.059	-1.164	1.071	7271.97
mafAR_expanding	-0.363	-1.568	0.848	6487.17
mafAR_rolling	-0.053	-1.191	1.059	7028.66
mafSD_expanding	-0.891	-2.053	0.297	6050.84
mafSD_rolling	-0.585	-1.697	0.528	6910.91
maxAR_expanding	-0.38	-1.566	0.826	6352.85
maxAR_rolling	-0.07	-1.186	1.056	6830.69
maxCOV_expanding	-0.111	-1.287	1.099	6405.66
maxCOV_rolling	-0.316	-1.484	0.786	7160.19
maxSD_expanding	0.162	-1.05	1.407	6410.5
maxSD_rolling	0.732	-0.413	1.909	7128.34
meanAR_expanding	1.27	-0.103	2.827	6754.02
meanAR_rolling	-0.303	-1.45	0.816	6981.3
meanSD_expanding	-0.368	-1.559	0.831	5843.92
meanSD_rolling	1.377	0.152	2.725	6999.22
mutINFO_expanding	1.782	0.319	3.503	6973.31
mutINFO_rolling	0.179	-0.914	1.343	6927.06
pcaAR_expanding	0.487	-0.756	1.774	6604.05
pcaAR_rolling	0.452	-0.666	1.617	6921.03
pcaSD_expanding	0.837	-0.467	2.238	5502.2
pcaSD_rolling	-0.563	-1.746	0.558	6735.44
scaled_ML	1.226	0.113	2.389	6992.78
SD + skew_expanding	0.374	-0.551	1.135	4091.32
SD_expanding	-0.276	-1.194	0.499	4015.75
SD_rolling	0.431	-0.673	1.415	6261.03
skew_expanding	0.828	-0.094	1.612	4259.85
skew_rolling	0.124	-0.986	1.075	6312
unscaled_ML	-1.412	-2.547	-0.24	6962.31

Table S13. Raw coefficient estimates for the influence of each early warning signal indicator on the correct classification of non-transitioning yearly lake plankton data. Rhat was equal to 1 for all estimates.

Early warning signal indicator	Coefficient estimate (median)	Lower 95% credible interval	Upper 95% credible interval	Effective sample size
ar1 + SD_expanding	-0.037	-0.999	0.926	6441.52
ar1 + skew_expanding	0.382	-0.619	1.366	6465.21
ar1_expanding	-0.116	-1.066	0.848	6473.86
ar1_rolling	-0.297	-1.583	1.01	6755.38
eigenCOV_expanding	0.564	-1.275	2.659	6898.07
eigenCOV_rolling	-0.449	-1.663	0.779	6817.43
eigenMAF_expanding	0.57	-1.273	2.675	7059.78
eigenMAF_rolling	0.1	-1.091	1.322	7481.42
mafAR_expanding	-1.572	-3.081	0.08	7372.55
mafAR_rolling	-0.16	-1.337	1.026	6774.67
mafSD_expanding	-0.302	-2.068	1.52	7182.36
mafSD_rolling	-1.01	-2.256	0.194	6888.93
maxAR_expanding	-0.33	-2.032	1.62	6821.15
maxAR_rolling	1.011	-0.243	2.312	7242.09
maxCOV_expanding	0.581	-1.29	2.663	7160.89
maxCOV_rolling	-0.435	-1.693	0.728	7186.74
maxSD_expanding	0.563	-1.315	2.625	7363.51
maxSD_rolling	1.736	0.404	3.229	6380.13
meanAR_expanding	-0.308	-2.03	1.581	7132.62
meanAR_rolling	0.388	-0.801	1.638	7136.08
meanSD_expanding	-0.319	-1.983	1.582	7150
meanSD_rolling	1.338	0.076	2.735	7260.66
mutINFO_expanding	0.596	-1.326	2.668	7558.13
mutINFO_rolling	-2.519	-4.065	-1.097	7382.04
pcaAR_expanding	0.588	-1.302	2.645	6587.8
pcaAR_rolling	0.386	-0.827	1.643	7164.39
pcaSD_expanding	0.555	-1.286	2.651	7363.95
pcaSD_rolling	-0.442	-1.656	0.745	6794.44
scaled_ML	3.496	1.78	5.066	6548.54
SD + skew_expanding	-0.265	-1.198	0.696	6487.91
SD_expanding	0.229	-0.739	1.231	6386.85
SD_rolling	0.298	-0.977	1.603	6704.27
skew_expanding	0.703	-0.261	1.687	6547.69
skew_rolling	-0.052	-1.334	1.248	6792.27
unscaled_ML	-2.796	-4.467	-1.21	6776.58
